# Meta-analysis of efficacy of probiotics in reducing postoperative infections and improving outcomes in gastrointestinal surgery

**DOI:** 10.3389/fsurg.2026.1746191

**Published:** 2026-05-08

**Authors:** Muhammad Shamim

**Affiliations:** Department of Surgery, College of Medicine, Prince Sattam bin Abdulaziz University, Alkharj, Saudi Arabia

**Keywords:** ERAS protocol, gastrointestinal surgery, postoperative infections, probiotics, surgical wound infections, lactobacillus species, bifidobacterium species

## Abstract

**Background:**

Postoperative infections remain a major cause of morbidity and mortality following gastrointestinal surgery. This meta-analysis aims to evaluate the efficacy of perioperative probiotic use in reducing postoperative infections (POI).

**Methods:**

Eight randomized controlled trials (RCTs) with 755 participants were included. The effect size was calculated as the Odds Ratio (OR) and Risk Ratio (RR) using a random-effects model.

**Results:**

The pooled analysis suggested a potential benefit in reducing overall postoperative infections [OR: 0.57 (95% CI: 0.32–1.01); *P* = 0.057] and wound infection [OR: 0.61 (95% CI: 0.35–1.06); *P* = 0.073]. While the overall effect narrowly missed statistical significance, subgroup analysis revealed a statistically significant reduction in POI when multi-strain *Lactobacillus* and *Bifidobacterium* formulations were used [RR: 0.64 (95% CI: 0.49–0.83)]. The absolute risk reduction (ARR) for this multi-strain subgroup was 13.5%, corresponding to a highly favorable number needed to treat (NNT) of 7.

**Conclusion:**

The current evidence regarding the overall efficacy of perioperative probiotics in decreasing postoperative infectious complications narrowly misses statistical significance. However, the targeted use of multi-strain *Lactobacillus* and *Bifidobacterium* formulations significantly reduces the risk of infectious complications. These specific regimens offer a clinically meaningful benefit and support its inclusion in Enhanced Recovery After Surgery (ERAS) protocols for patients undergoing major gastrointestinal surgery.

## 1 Introduction

Postoperative infections (POI) remain a major cause of morbidity and mortality, and consequently increased healthcare costs following gastrointestinal surgery (1, 2). Despite advancements in surgical techniques, antibiotic prophylaxis and Enhanced Recovery After Surgery (ERAS) protocols, the incidence of surgical site infections, pneumonia and intra-abdominal abscesses remain significant (3, 4). The gut microbes, often called as “forgotten organ”, plays a critical role in the pathogenesis of POI (5). Surgical stress, prolonged fasting, antibiotic administration and bowel preparation can lead to gut dysbiosis, characterized by decrease in the number of beneficial bacteria and an increase of potential pathogens (6, 7). This dysbiosis compromises the intestinal barrier function and promotes bacterial translocation, which subsequently triggers systemic inflammation and infection (8, 9).

Probiotics, defined as live microorganisms that, when administered in adequate amounts, confer a health benefit on the host, have emerged as a potential therapeutic strategy to mitigate these risks (10). By restoring gut microbes, strengthening the intestinal mucosal barrier and modulating the host immune response, the perioperative probiotic administration is hypothesized to reduce the incidence of POI (11). Several systematic reviews and meta-analyses have previously investigated the role of probiotics in preventing postoperative infections in patients undergoing gastrointestinal surgery (1, 2). While some have reported promising results, the evidence remains inconclusive due to variations in study design, probiotic formulations, and patient populations. This updated systematic review and meta-analysis aims to provide a more current and comprehensive synthesis of the evidence by including recently published randomized controlled trials (RCTs). Hence, the objective of this study is to evaluate the effect of perioperative probiotic administration on the incidence of overall postoperative infective complications and specific outcomes, such as wound infection, in patients undergoing gastrointestinal surgery.

## 2 Methods

This quantitative systemic review and meta-analysis was conducted in accordance with the Preferred Reporting Items for Systemic Reviews and Meta-Analysis (PRISMA) guidelines.

### 2.1 Search strategy and study selection

A comprehensive search was performed on October 4, 2025, across major electronic databases including PubMed, Embase and the Cochrane Central Register of Controlled Trials (CENTRAL). To ensure no recent publications were missed prior to manuscript submission, an updated search was performed on November 12, 2025. The search strategy combined both keywords (e.g., probiotics, surgical patients, postoperative infection) and Medical Subject Headings (MeSH) terms (e.g., probiotics, synbiotics, Lactobacillus, Bifidobacterium, colorectal surgery, gastrointestinal surgery, hepatectomy, pancreatoduodenectomy, surgical site infection). While our search was designed to capture the most commonly studied probiotic strains, we acknowledge that it may not have comprehensively captured studies using alternative probiotic organisms like *Bacillus*, *Enterococcus*, *Streptococcus* or *Escherichia coli*. This represents a potential limitation that should be addressed in future systemic reviews. No language restrictions were applied. We also searched for gray literature in clinical trial registries. The search was limited to studies published between 2014 and 2025. This time frame was chosen to ensure that the included studies reflect contemporary surgical practices and the standardized implementation of ERAS protocols, thereby increasing the homogeneity of the included studies and the relevance of the findings to current clinical practice.

The complete retrieval formulas for each database were as follows:

**PubMed/MEDLINE:** (((“Probiotics”[Mesh] OR “Synbiotics”[Mesh] OR “Lactobacillus”[Mesh] OR “Bifidobacterium”[Mesh] OR “Saccharomyces”[Mesh]) OR (probiotic*[Title/Abstract] OR symbiotic*[Title/Abstract] OR lactobacillus[Title/Abstract] OR bifidobacterium[Title/Abstract] OR saccharomyces[Title/Abstract])) AND ((“Digestive System Surgical Procedures”[Mesh] OR “Colorectal Surgery”[Mesh] OR “Hepatectomy”[Mesh] OR “Pancreaticoduodenectomy”[Mesh] OR (“gastrointestinal surgery”[Title/Abstract] OR “colorectal surgery”[Title/Abstract] OR “abdominal surgery”[Title/Abstract] OR “hepatectomy”[Title/Abstract] OR “pancreaticoduonectomy”[Title/Abstract] OR “bowel surgery”[Title/Abstract])) AND ((“Surgical Wound Infection”[Mesh] OR “Postoperative Complications”[Mesh]) OR (“postoperative infection*”[Title/Absract] OR “surgical site infection*”[Title/Abstract] OR “wound infection*”[Title/Abstract] OR “infectious complication*”[Title/Abstract] OR POI[Title/Abstract]))) AND ((randomized controlled trial[Publication Type] OR randomized[Title/Abstract] OR placebo[Title/Abstract)).

**Embase:** (“probiotic agent”/exp OR “synbiotic agent”/exp OR “Lactobacillus”/exp OR “Bifidobacterium”/exp OR “Saccharomyces”/exp OR probiotic*:ab,ti OR synbiotic*:ab,ti OR lactobacillus:ab,ti OR bifidobacterium:ab,ti OR saccharomyces:ab,ti) AND (“digestive system surgery”/exp OR “colorectal surgery”/exp OR “liver resection”/exp OR “pancreaticoduodenectomy”/exp OR “gastrointestinal surgery”:ab,ti OR “colorectal surgery”:ab,ti OR “abdominal surgery”:ab,ti OR “hepatectomy”:ab,ti OR “pancreatoduodenectomy”:ab,ti OR “bowel surgery”:ab,ti) AND (“surgical infection”/exp OR “postoperative complication”/exp OR “postoperative infection*”:ab,ti OR “surgical site infection*”:ab,ti OR “wound infection*”:ab,ti OR “infectious complication*”:ab,ti OR POI:ab,ti) AND (“randomized controlled trial”/exp OR randomized:ab,ti OR placebo:ab,ti).

**Cochrane Central Register of Controlled Trials (CENTRAL)**: ([mh Probiotics] OR [mh Synbiotics] OR [mh Lactobacillus] OR [mh Bifidobacterium] OR [mh Saccharomyces] OR probiotic*:ti,ab,kw OR synbiotic*:ti,ab,kw OR lactobacillus:ti,ab,kw OR bifidobacterium:ti,ab,kw OR saccharomyces:ti,ab,kw) AND ([mh “Digestive System Surgical Procedures”] OR [mh “Colorectal Surgery”] OR [mh Hepatectomy] OR [mh Pancreaticoduodenectomy] OR “gastrointestinal surgery”:ti,ab,kw OR “colorectal surgery”:ti,ab,kw OR “abdominal surgery”:ti,ab,kw OR “hepatectomy”:ti,ab,kw OR “pancreatoduodenectomy”:ti,ab,kw OR “bowel surgery”:ti,ab,kw) AND ([mh “Surgical Wound Infection”] OR [mh “Postoperative Complications”] OR “postoperative infection*”:ti,ab,kw OR “surgical site infection*”:ti,ab,kw OR “wound infection*”:ti,ab,kw OR “infectious complication*”:ti,ab,kw OR POI:ti,ab,kw).

In addition to the electronic database searches, a manual search of the reference lists of included studies and relevant review articles was performed to identify any additional eligible trials. To minimize publication bias, a gray literature search was conducted using the following clinical trial registries:

- ClinicalTrials.gov (https://clinicaltrials.gov/)
- WHO International Clinical Trials Registry Platform (ICTRP) (https://www.who.int/clinical-trials-registry-platform)
- European Union Clinical Trials Register (EudraCT) (https://www.clinicaltrialsregister.eu/)

The initial search resulted in **1,501** records. Limiting the search to studies published between 2014 and 2025 resulted in **1,168** studies. After removing duplicates, screening titles and extracts, and excluding studies without data, **246** studies were retained for full-text review. Of these, **49** were identified as randomized controlled trials (RCTs). A total of **41** studies were excluded at this stage, due to different primary and/or secondary outcomes (e.g., focusing solely on gut permeability markers without clinical infection data). Finally, **8** RCTs were selected for inclusion in the quantitative synthesis (meta-analysis).

### 2.2 Eligibility criteria

#### 2.2.1 Inclusion criteria

Only parallel-group RCTs comparing perioperative administration of probiotics or synbiotics (probiotics plus prebiotics) vs. placebo or standard care were included. The study population was restricted to adult patients (≥18 years) undergoing elective abdominal or gastrointestinal surgery. Elective surgery was defined as planned surgical procedures scheduled in advance, performed under controlled conditions, with adequate preoperative preparation.

#### 2.2.2 Exclusion criteria

Studies were excluded if they met any of the following criteria: non-randomized studies, observational studies, case reports, or reviews; studies involving pediatric populations; studies where probiotics were administered exclusively postoperatively; or studies lacking sufficient data to calculate odds ratios (OR) and 95% confidence intervals (CI). Furthermore, to ensure homogeneity of the patient population and to allow for adequate perioperative probiotic administration as per study protocols, emergency surgeries (defined as unplanned procedures performed within 24 h of admission due to acute conditions such as perforation, obstruction or acute bleeding) were excluded from this analysis.

#### 2.2.3 Patient exclusion criteria for comorbidities

The included studies applied variable exclusion criteria for patients with comorbidities that might increase infection risk or affect probiotic efficiency. Common exclusion criteria across the included RCTs were:

- Immunodeficiency or current immunosuppressive therapy
- Uncontrolled or severe diabetes mellitus (e.g., HbA1c > 8%)
- Severe hepatic or renal dysfunction
- Active malignancy
- Known allergy or contraindication to the study probiotics or antibiotics
- Pregnancy or lactation

The heterogeneity in comorbidity exclusion criteria across the primary studies represents a potential source of between-study heterogeneity in our meta-analysis.

#### 2.2.4 Focus on lower gastrointestinal and hepatic surgery

Although the search was not restricted to specific types of gastrointestinal surgery, the final analysis focused on lower gastrointestinal and hepato-pancreatic surgeries. Upper gastrointestinal surgeries (esophageal, gastric and small bowel) were underrepresented in the available literature meeting our inclusion criteria. This focus reflects the current evidence base, where probiotics have been more extensively studies in colorectal surgery due to the greater bacterial load in the colon and the established role of colonic dysbiosis in postoperative complications. Additionally, the mechanism of probiotic action, especially the restoration of colonic microbiota and enhancement of intestinal barrier function, are most relevant to lower GI and hepatic surgeries. Future research should investigate the efficacy of probiotics in upper GI surgery, where the evidence remains limited.

### 2.3 Data extraction and risk of bias assessment

Two independent reviewers (ML and HM) extracted data on study characteristics (author, year, patient population, intervention, control), sample size and outcome data (number of events and total participants in each group) for the calculation of effect sizes. Any discrepancies between the reviewers were resolved through discussion and consensus, or by consulting a third reviewer (MS).

The methodological quality and risk of bias for each included study were assessed using the Cochrane Collaboration's Risk of Bias tool (RoB 1.0) across seven domains: random sequence generation, allocation concealment, blinding of participants and healthcare providers, blinding of outcome assessment, incomplete outcome data, selective reporting and other bias. The detailed judgment criteria for each domain were as follows:

- Random Sequence Generation (Selection Bias):
  - *Low risk*: Explicit description of a valid randomization method (e.g., computer-generated random numbers, random number table, coin flip).
  - *High risk*: Use of a non-random method (e.g., alternation, case record number, date of birth).
  - *Unclear risk*: Randomization mentioned, but the specific method was not described.
- Allocation Concealment (Selection Bias):
  - *Low risk*: Use of methods preventing foreknowledge of treatment assignment (e.g., central allocation, sequentially numbered opaque sealed envelopes).
  - *High risk*: Allocation sequence known or predictable before enrollment (e.g., open random allocation schedule, unsealed envelopes).
  - *Unclear risk*: Concealment mentioned, but the specific method was not described.
- Blinding of Participants and Personnel (Performance Bias):
  - *Low risk*: Double-blind design with an identical placebo, or open-label design where the outcome is not likely to be influenced by lack of blinding.
  - *High risk*: Open-label design or obvious differences between interventions where the outcome is likely to be influenced by lack of blinding.
  - *Unclear risk*: Blinding mentioned, but the specific method or extent was not described.
- Blinding of Outcome Assessment (Detection Bias):
  - *Low risk*: Outcome assessors were blinded to the intervention assignment.
  - *High risk*: Outcome assessors were aware of the intervention assignment.
  - *Unclear risk*: Blinding of outcome assessors was not mentioned.
- Incomplete Outcome Data (Attrition Bias):
  - *Low risk*: No missing outcome data, or dropout rate <10% with balanced dropout between groups and reasons provided.
  - *High risk*: Dropout rate >10%, imbalanced dropout between groups, or reasons for missing data likely related to true outcomes.
  - *Unclear risk*: Dropout rate or reasons for missing data were not reported.
- Selective Reporting (Reporting Bias):
  - *Low risk*: All pre-specified primary and secondary outcomes mentioned in the methods section were reported in the results.
  - *High risk*: Outcomes mentioned in the methods were not reported, or additional outcomes were reported without prior specification.
  - *Unclear risk*: Study protocol was not available for comparison.
- Other Bias:
  - *Low risk*: No other apparent sources of bias identified.
  - *High risk*: Presence of funding bias, conflict of interest, baseline imbalance, or other significant methodological concerns.
  - *Unclear risk*: Insufficient information to assess whether an important risk of bias exists.

### 2.4 Statistical analysis

The primary outcome measure was the incidence of overall postoperative infections, whereas secondary outcome measure was the incidence of wound infection. The meta-analysis was performed using a random-effects model, given the expected heterogeneity in patient populations, surgical procedures and probiotic strains. The effect size was expressed as the Odds Ratio (OR) with 95% Confidence Intervals (CI) for dichotomous outcomes. Heterogeneity was assessed using the Cochrane's *Q-*test and the *I*^2^ statistic, with *I*^2^ > 50% indicating substantial heterogeneity. The *P*-value of the *Q*-test was reported alongside the *I*^2^ statistic to provide a comprehensive assessment of heterogeneity. A two-sided *P*-value < 0.05 was considered statistically significant.

To explore potential sources of heterogeneity and examine the consistency of probiotic effects across different clinical contexts, pre-specified subgroup analyses were planned. These analyses were stratified by the type of surgery (colorectal vs. hepatic/pancreatic), probiotic formulation (single-strain vs. multi-strain/synbiotics), and duration of administration (preoperative vs. perioperative).

To translate the odds ratio into clinically meaningful terms, we calculated the absolute risk reduction (ARR) and number needed to treat (NNT) based on baseline infection risk estimates from the control groups of the included studies.

### 2.5 Publication bias assessment

Publication bias was not formally assessed using funnel plot asymmetry tests (Egger's test or Begg's test) due to the limited number of included studies (*n* = 8). Current methodological guidelines recommend a minimum of 10 studies for adequate statistical power in formal publication bias testing. With fewer than 10 studies, these tests have insufficient power to reliably distinguish publication bias from true heterogeneity or other sources of asymmetry.

However, we acknowledge that visual inspection of study effect sizes and sample sizes could provide exploratory information about potential asymmetry. We recommend that future meta-analyses with a larger number of studies perform formal publication bias assessments using established statistical methods. As a proxy for publication bias risk, we assessed the inclusion of gray literature, funding sources, and potential conflicts of interest across the included studies.

## 3 Results

### 3.1 Study selection and characteristics

**Figure 1:**
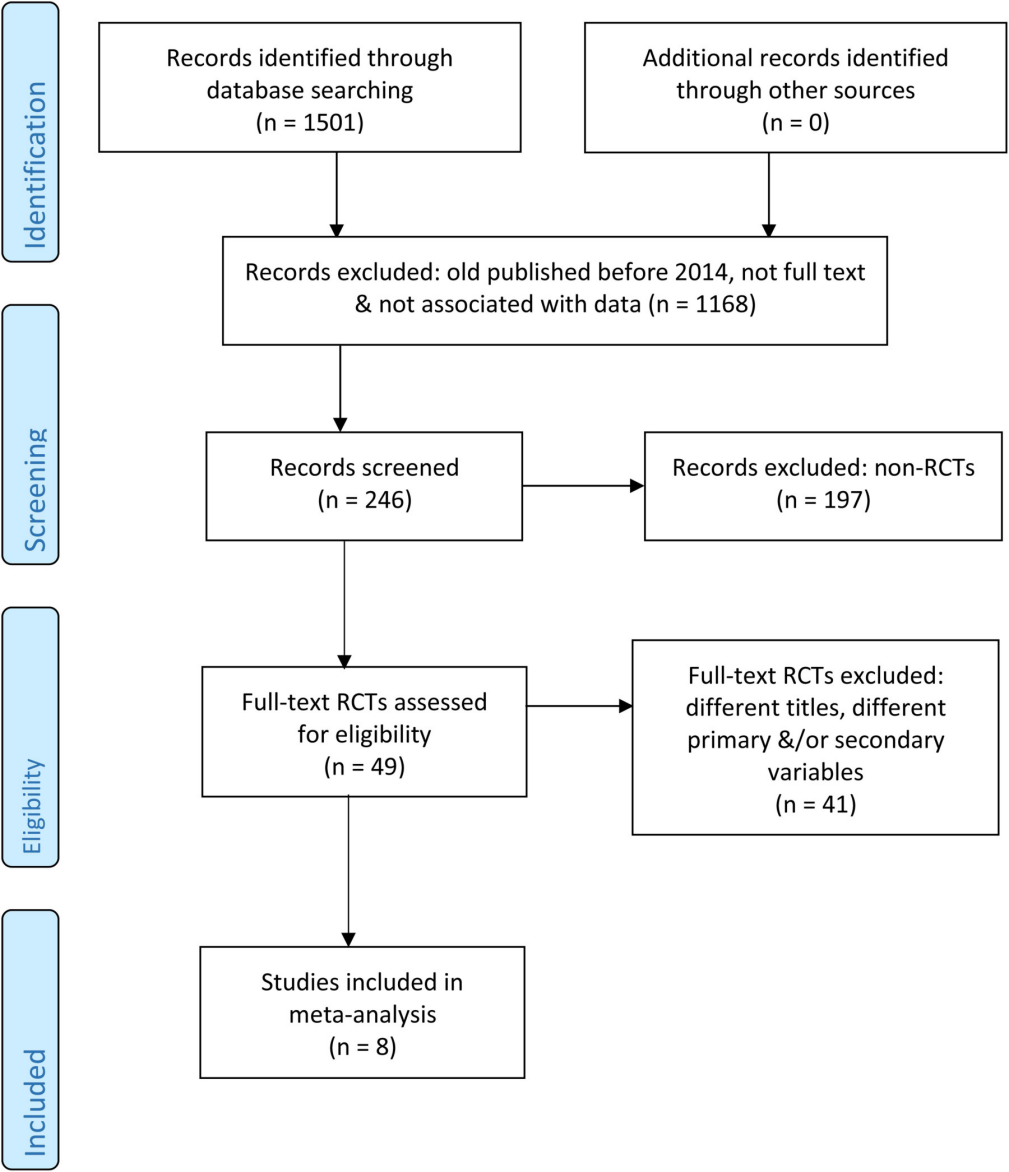
PRISMA flow diagram.

The details of study selection procedure are presented in PRISMA flow chart (Figure 1). A total of eight RCTs published between 2014 and 2022 were included in the final analysis, consisting of 755 patients (383 in the probiotic group and 372 in the control group). The studies were conducted across multiple countries, including France, Japan, Brazil, Malaysia and Greece. The pooled mean age of the patients was 64.8 years, with a male-to-female ration of 424:331 (56.2% male). The studies comprised different surgery types, including colorectal surgery (5 studies), liver resection (2 studies) and pancreatic surgery (1 study), reflecting the diverse application of probiotics in the perioperative setting. The study characteristics is presented in Table 1.

**Table 1:** Study characteristics.

Study (First Author, Year)	Total Patients (*N*)	Surgical Type	Age (P)	Age (C)	Sex M, F (P)	Sex M, F (C)
Roussel (12)	54	Liver resection	66	66.9	23, 4	23, 4
Yokoyama (13)	44	Pancreatoduodenectomy	65	65	6, 16	6, 16
Flesch (14)	91	Colorectal surgery	64.5	61.1	18, 31	19, 23
Tan (15)	40	Colorectal surgery	64.3	68.4	11, 9	13, 7
Consoli (16)	33	Colorectal surgery	51	59	10, 5	10, 8
Kotzampassi (17)	164	Colorectal surgery	65.9	66.4	57, 27	58, 22
Liu (18)	134	Liver surgery	65.62	60.16	35, 31	35, 33
Sadahiro (19)	195	Colorectal surgery	67	66	49, 51	51, 44

Table 2 shows characteristics of probiotic interventions. Most trials reported multi-strain formulations containing *Lactobacillus* and *Bifidobacterium* species, administered at doses of approximately 10^9^ colony-forming units (CFU) per dose. The duration of administration ranged from 7 days preoperatively up to 14 days postoperatively.

**Table 2:** Characteristics of probiotic interventions.

Study (Author, Year)	Probiotic Strain(s)	Dose	Duration	Control
Roussel (12)	*Lactobacillus* & *Bifidobacterium* strains	10⁹ CFU per dose	14 days preoperative	Placebo
Yokoyama (13)	*Lactobacillus* & *Bifidobacterium* strains	Not specified	5 days preoperative & 14 days Postoperative	No placebo
Flesch (14)	*Lactobacillus* & *Bifidobacterium* strains	10^9^ CFU per dose	Not specified	Placebo
Tan (15)	*Lactobacillus* & *Bifidobacterium* strains	3 × 10^10^ CFU per dose	7 days prior to surgery	Placebo
Consoli (16)	*Saccharomyces boulardii*	10⁹ CFU per dose	7 days prior to surgery	No placebo
Kotzampassi (17)	*Lactobacillus*, *Bifidobacterium* & *S. boulardii* strains	10⁹ CFU per dose	1 day preoperative & 14 days Postoperative	Placebo
Liu (18)	*Lactobacillus* & *Bifidobacterium* strains	2.6 × 10^14^ CFU per dose	6 days preoperative & 10 days Postoperative	Placebo
Sadahiro (19)	*Bifidobacterium bifidum*	Not specified	7 days preoperative & 5–10 days Postoperative	No placebo

**Figure 2:**
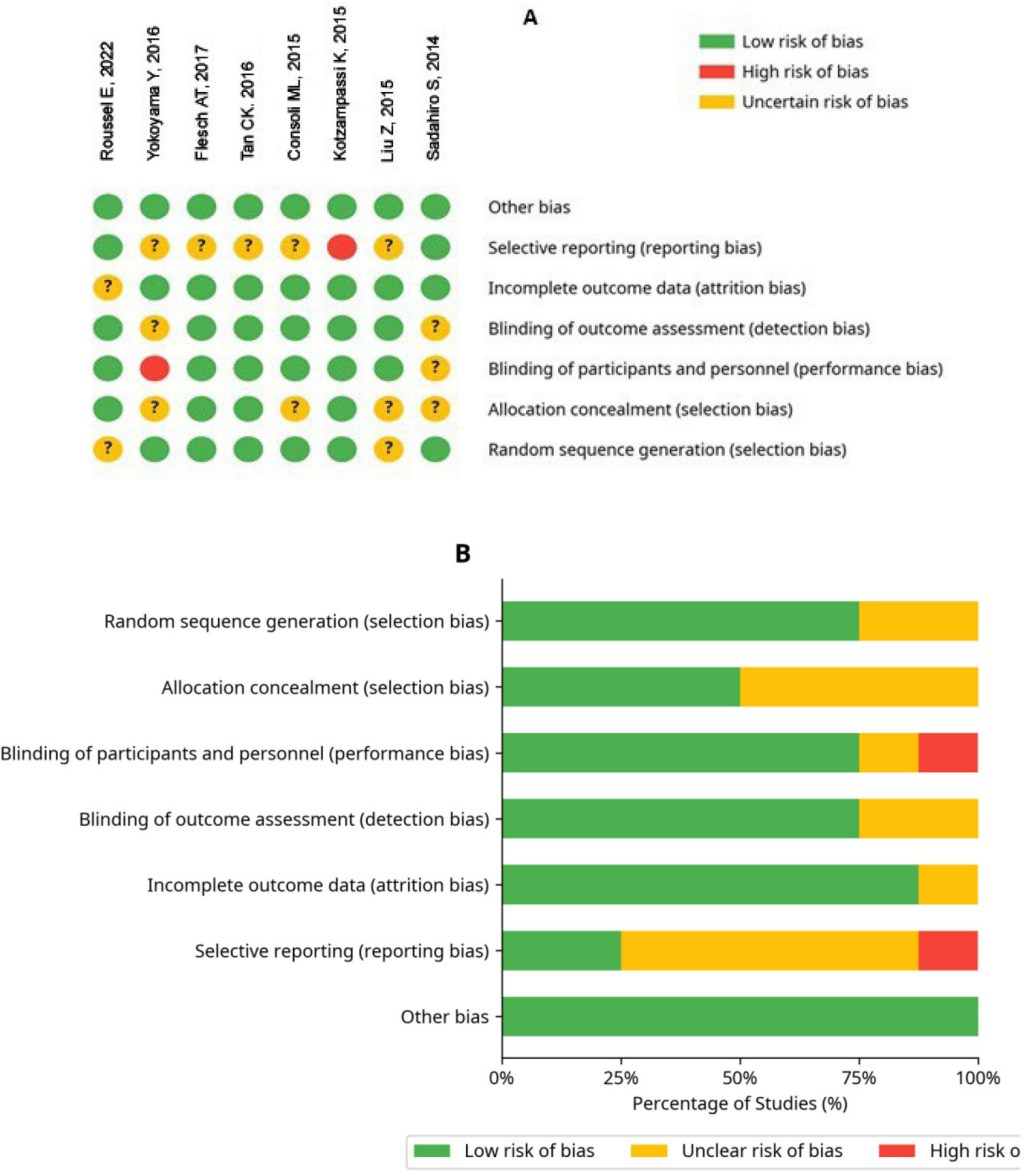
**(A)** risk of bias summary (dot plot). **(B)** Risk of bias graph (summary bar chart).

The methodological quality of the included studies is summarized in the figure below (Figures 2A,B). The assessment revealed a generally moderate risk of bias. The key findings are as follows:

- Blinding of participants & personnel: One study (Yukihiro Y, 2016) was judged to be at high risk due to a lack of blinding & placebo use.
- Allocation concealment: 4 studies were judged as unclear risk due to insufficient reporting of the method used to conceal the allocation sequence.
- Selective reporting: One study (Katerina K, 2015) was judged to be at high risk due to premature cessation based on an interim analysis for efficacy.

### 3.2 Primary and secondary outcomes

**Figure 3:**
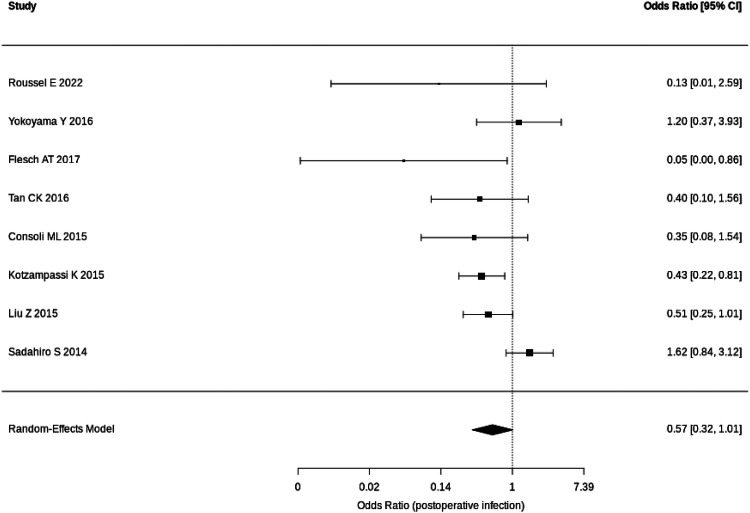
Forest plot for overall postoperative infective complications.

**Figure 4:**
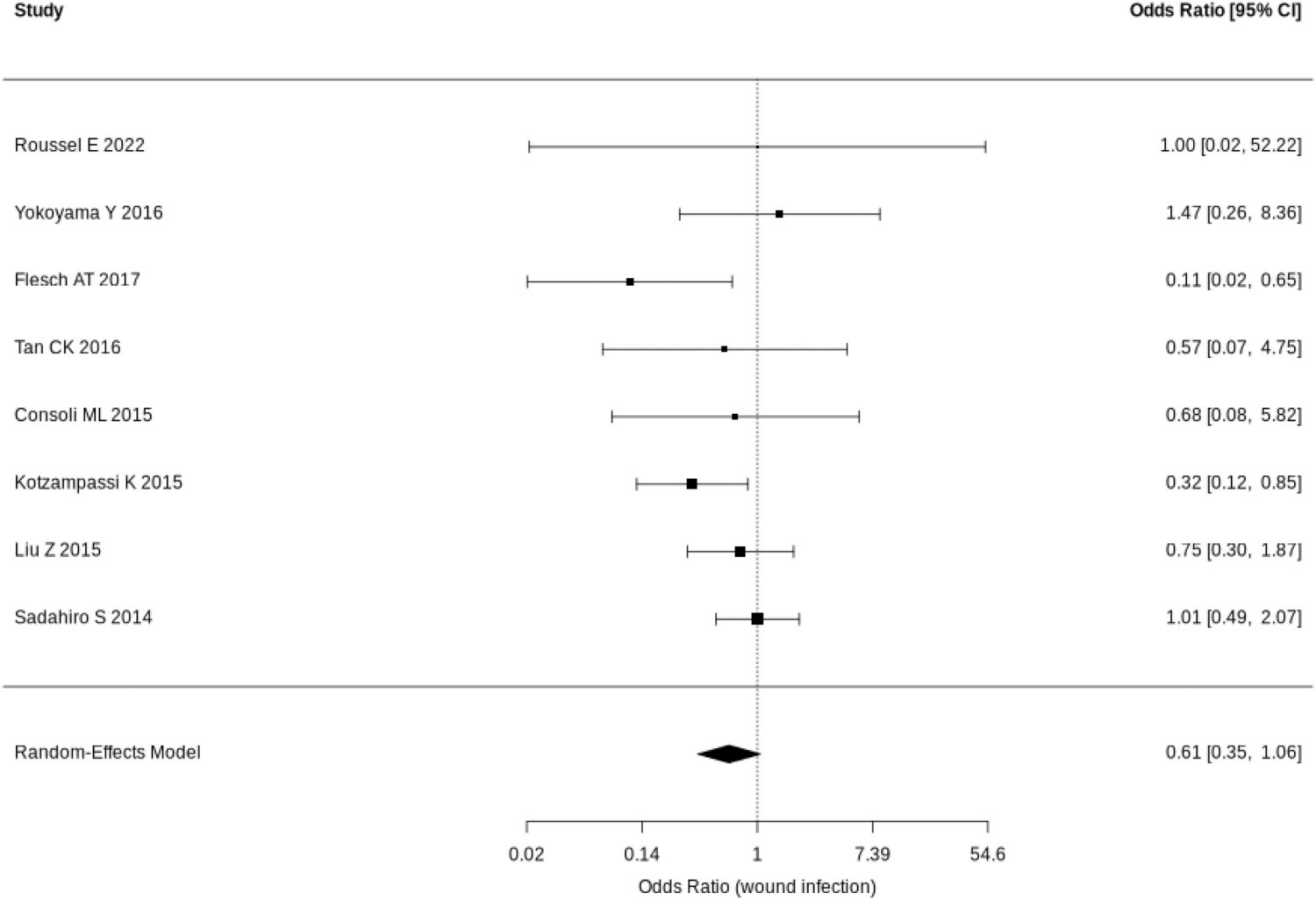
Forest plot for wound infection.

The results of meta-analysis of our primary outcome measure (overall postoperative infective complications) and secondary outcome measure (wound infection) are shown in the forest plots (Figures 3, 4). Random-effects meta-analysis was performed.

The pooled analysis showed a non-significant trend toward reducing overall postoperative infective complications [OR: 0.57 (95% CI: 0.32–1.01); *P* = 0.057]. The corresponding Risk Ratio (RR) was 0.73 (95% CI: 0.51–1.05). The level of heterogeneity was moderate (*I*^2^ = 53%), suggesting a mixed effect of probiotics on the overall postoperative infective complications across different study settings. However, the *Q*-test was statistically significant (*P* = 0.034), pointing to meaningful between-study variability likely driven by differences in surgical type, probiotic regimen and patient populations.

The pooled analysis for wound infection also showed a non-significant protective trend [OR: 0.61 (95% CI: 0.35–1.06); *P* = 0.073]. The corresponding Risk Ratio (RR) was 0.67 (95% CI: 0.42–1.07). The level of heterogeneity was low-moderate (*I*^2^ = 24%), suggesting a more consistent effect of probiotics on this specific outcome across different study settings. However, for this outcome, the *Q*-test was not significant (*P* = 0.31) suggesting the studies were reasonably consistent for this specific outcome.

### 3.3 Subgroup analysis

**Figure 5:**
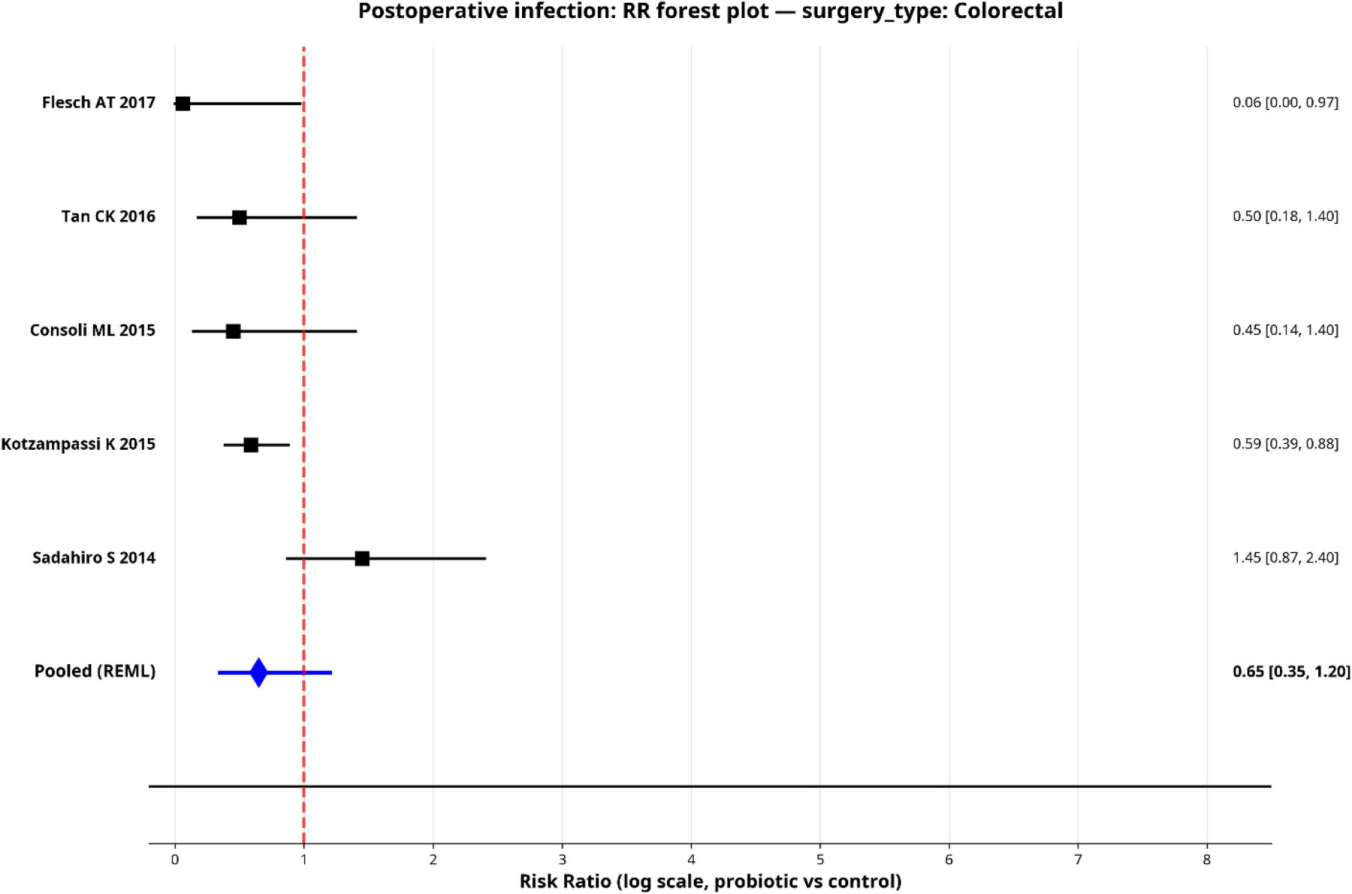
Forest plot for subgroup—colorectal surgery.

**Figure 6:**
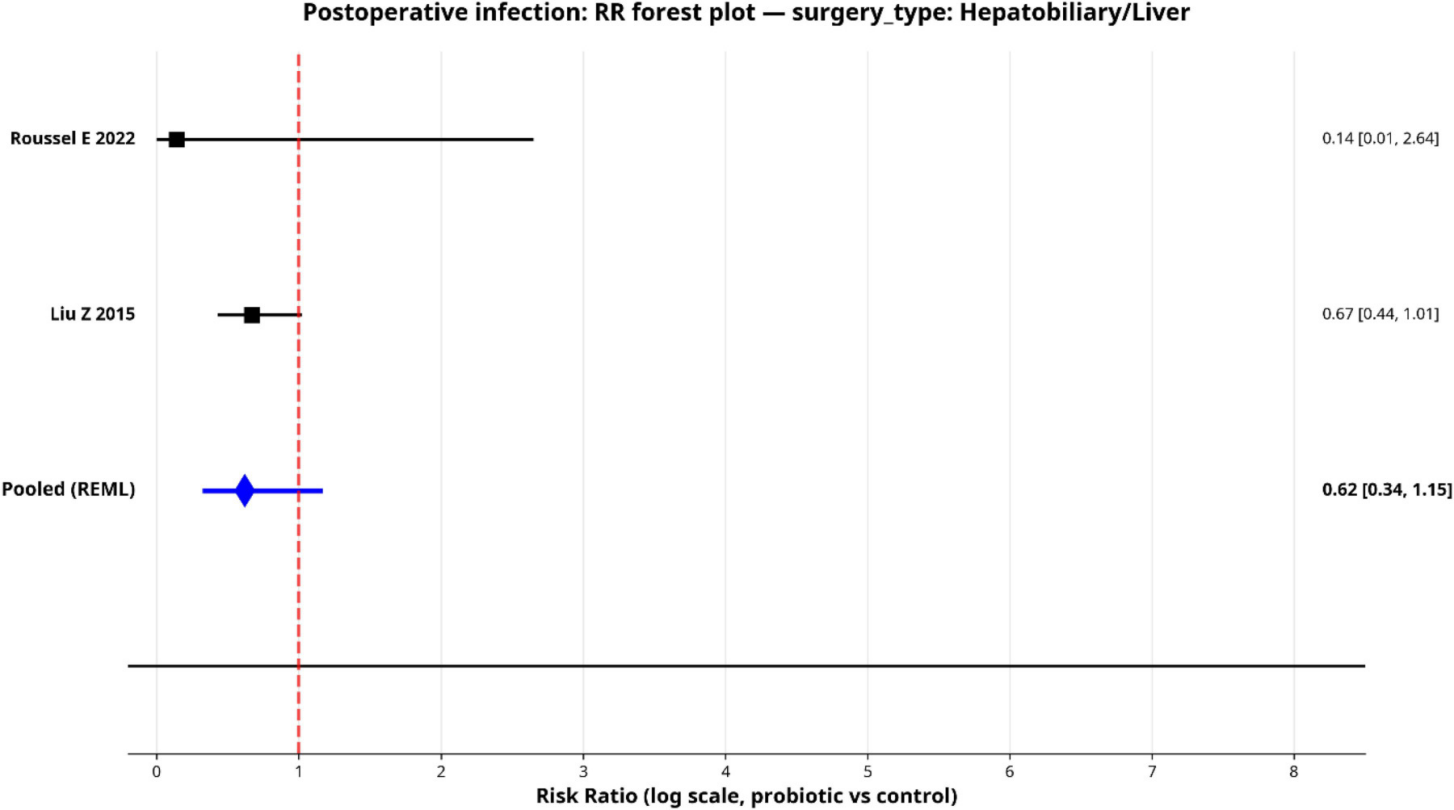
Forest plot for subgroup—hepatic surgery.

**Figure 7:**
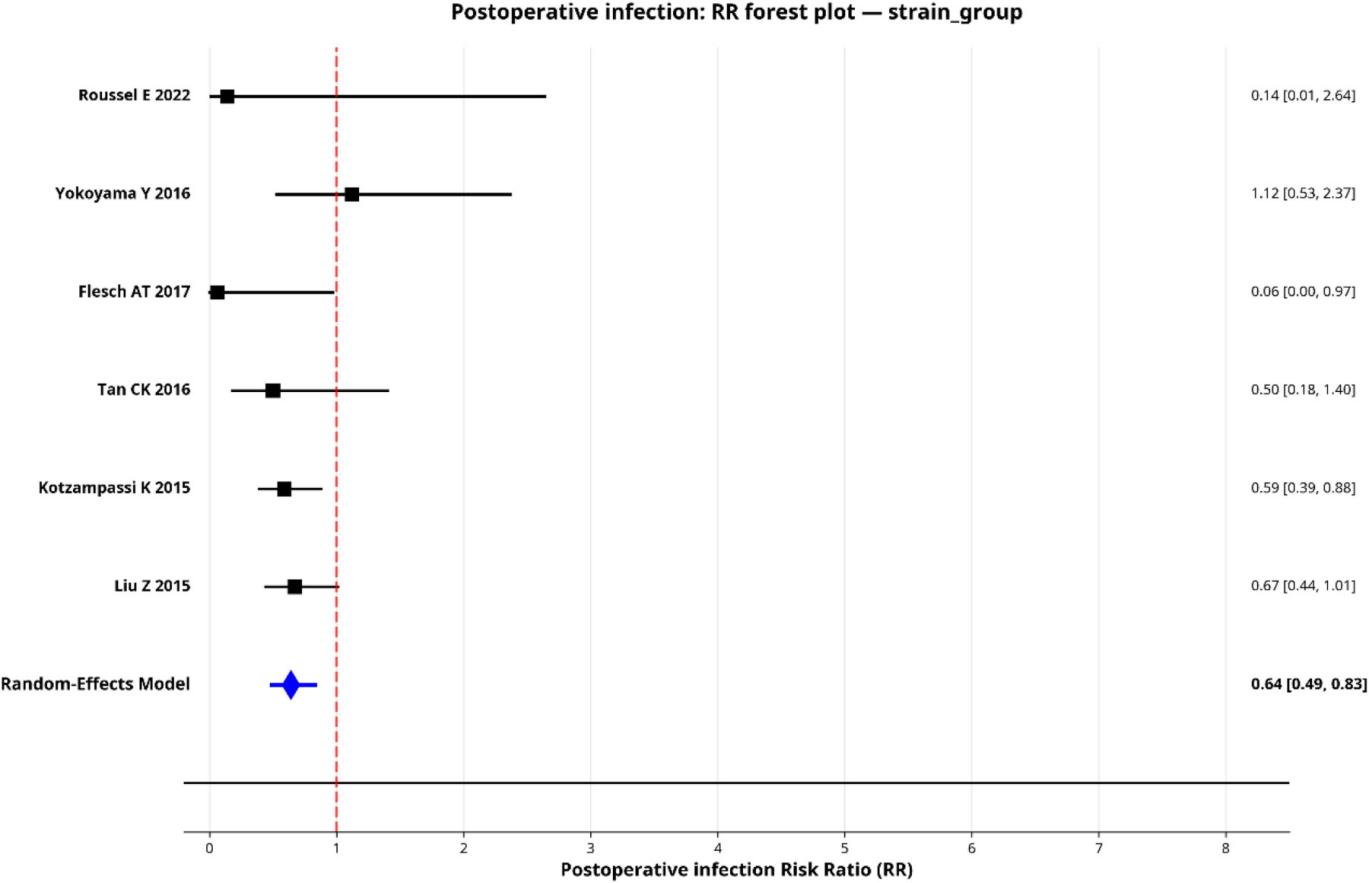
Forest plot for subgroup—probiotic strain.

**Figure 8:**
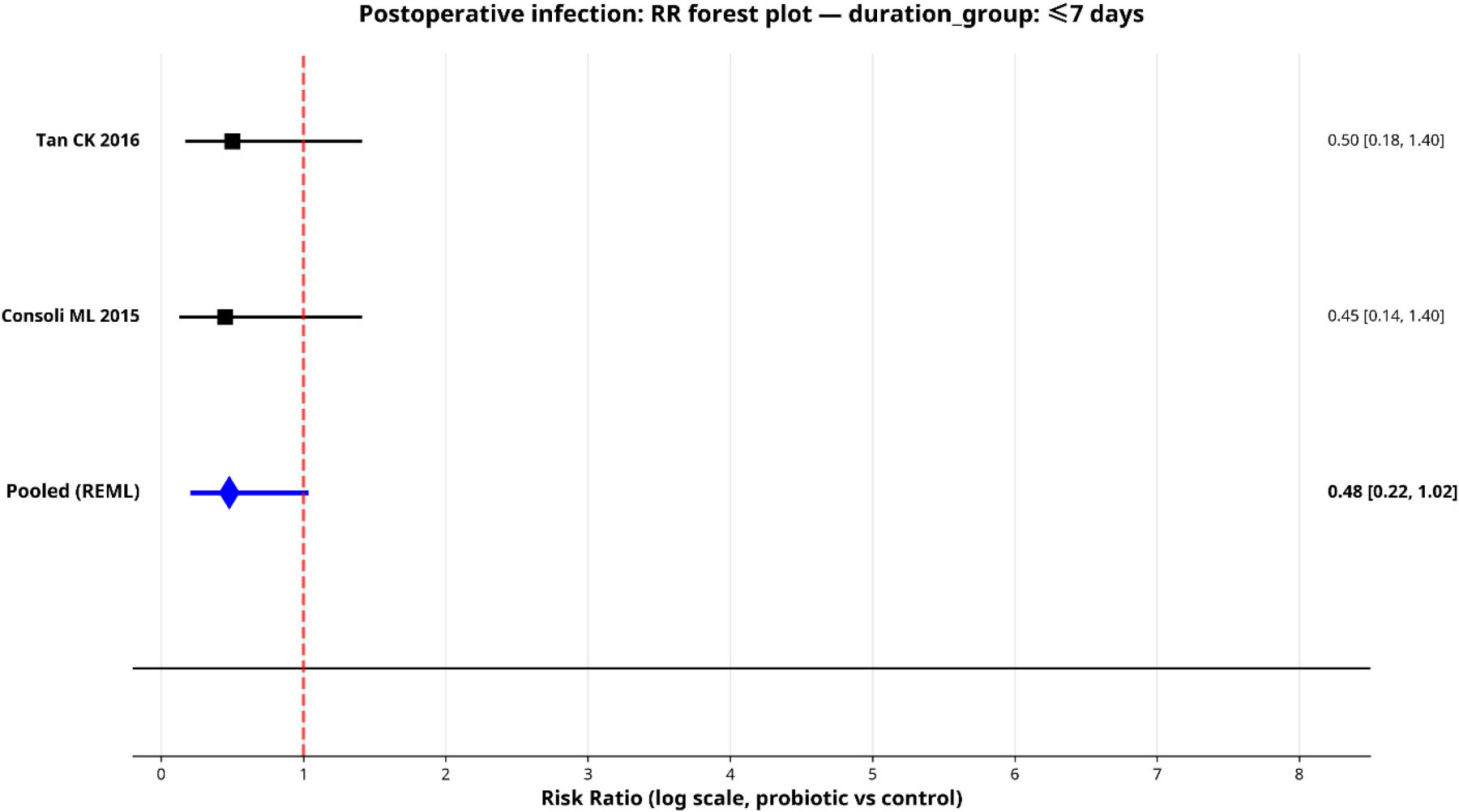
Forest plot for subgroup—short duration.

**Figure 9:**
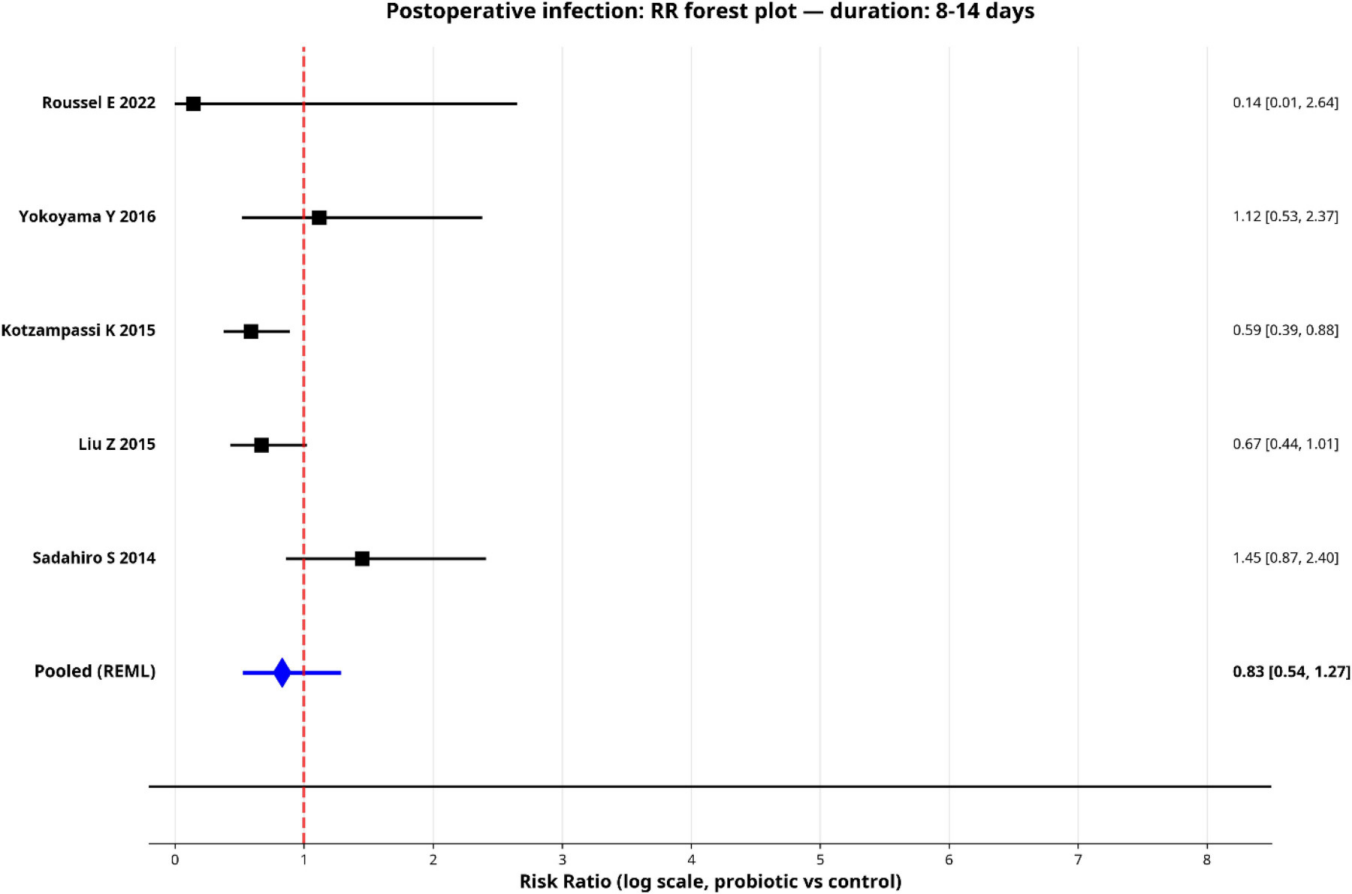
Forest plot for subgroup—long duration.

To explore sources of heterogeneity, we performed pre-specified subgroup analyses for postoperative infection by surgical type, probiotic strain and administration duration. Only subgroups with two or more studies were pooled, and the results are shown in forest plots (Figures 5–9).

#### 3.3.1 By surgery type

When stratified by surgery type, both subgroups showed directionally favorable effects, but neither reached statistical significance individually. The colorectal surgery subgroup (5 studies) yielded a pooled RR of 0.65 (95% CI: 0.35–1.20) with considerable heterogeneity (*I*^2^ = 65.1%). The hepatobiliary surgery subgroup (2 studies) yielded a pooled RR of 0.62 (95% CI: 0.34–1.15) and was more homogeneous (*I*^2^ = 4.8%).

#### 3.3.2 By probiotic strain

This analysis yielded the most striking finding. The subgroup utilizing multi-strain *Lactobacillus* + *Bifidobacterium* formulations (6 studies) demonstrated a statistically significant reduction in postoperative infections, with a pooled RR of 0.64 (95% CI: 0.49–0.83) and a near-zero heterogeneity (*I*^2^ ≈ 0%). Meta-regression confirmed that strain type was a significant moderator of effect (QM test *P* = 0.013), explaining all of the between-study variability (*R*^2^ = 100%).

#### 3.3.3 By regimen duration

Shorter-duration regimens (≤days; 2 studies) showed a larger point estimate of benefit [pooled RR: 0.48 (95% CI: 0.22–1.02); *I*^2^ = 0%], though this didn't reach statistical significance. The longer duration group (8–14 days; 5 studies) showed a more attenuated effect with higher heterogeneity [pooled RR: 0.83 (95% CI: 0.54–1.27); *I*^2^ = 61.0%].

### 3.4 Clinical implication and absolute risk reduction

**Figure 10:**
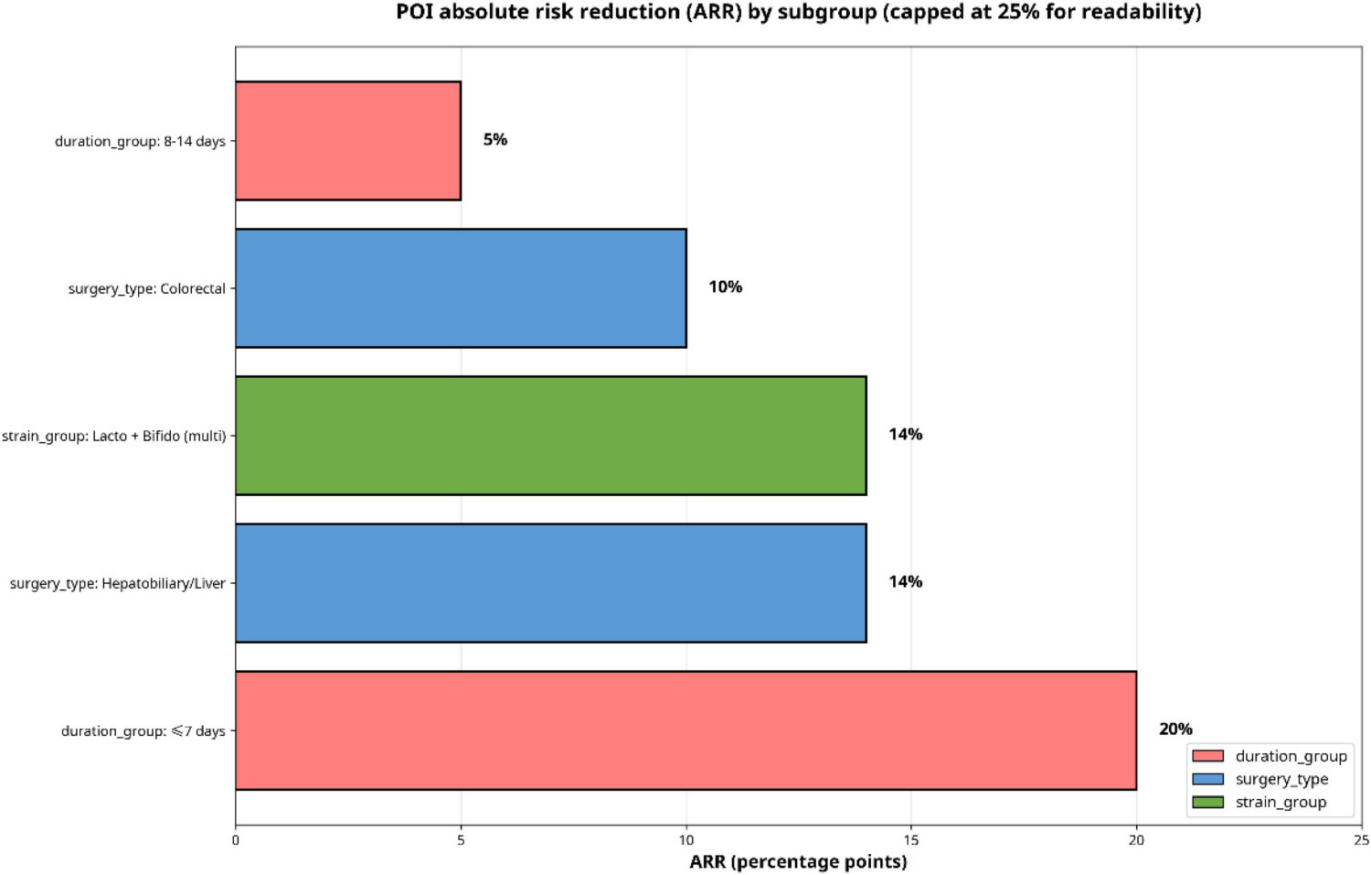
POI absolute risk reduction (ARR).

**Figure 11:**
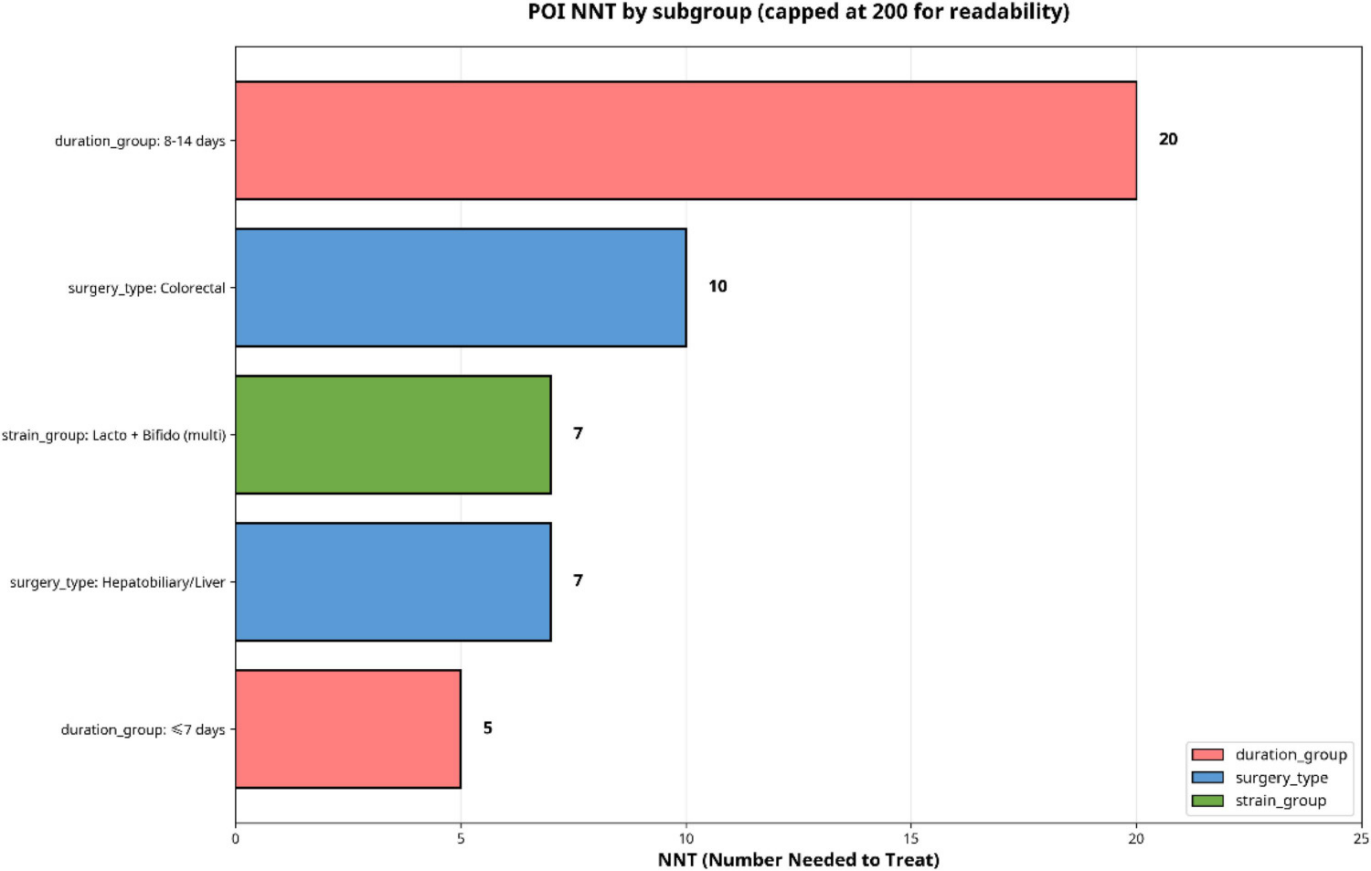
POI number needed to treat (NNT).

To quantify the potential real-world impact, absolute risk reduction (ARR) and number needed to treat (NNT) were derived from the pooled RR applied to the weighted baseline control-group infection rate within each subgroup. These are visualized in Figures 10, 11.

The most favorable clinical benefit emerged for multi-strain *Lactobacillus* + *Bifidobacterium* formulations. With a baseline control risk of 38.2% and a pooled RR of 0.64, the ARR was 13.5%. This corresponds to an NNT of approximately 7, meaning that treating 7–8 patients with this specific probiotic regimen would prevent one postoperative infection.

For the surgical subgroups, colorectal surgery (baseline risk 31.8%, RR 0.67) yielded an ARR of 10.6% and an NNT of ∼9. Hepatobiliary surgery (baseline risk 38.9%, RR 0.62) yielded an ARR of 14.6% and an NNT of ∼7.

Regarding administration duration, shorter regimen (≤ 7 days) demonstrated the most favorable NNT of ∼5 (baseline risk 42.1%, RR 0.48, ARR 20.6%), while longer regimens (8–14 days) yielded a less favorable NNT of ∼17 (baseline risk 35.3%, RR 0.83, ARR 6.0%).

## 4 Discussion

The findings of this meta-analysis suggest a potential benefit of perioperative probiotics in decreasing the incidence of overall postoperative infections as well as wound infection in patients undergoing gastrointestinal surgery. While the pooled Odds Ratio for overall POI (OR: 0.57) suggests a clinically meaningful reduction in risk; however, the 95% confidence interval crosses the line of no effect (OR = 1.0), with a *P*-value of 0.057. This finding, while statistically non-significant, may be limited by the small number of included studies and the inherent heterogeneity among them. The observed heterogeneity for the overall outcome (*I*^2^ = 53%) indicates that subgroup analysis (e.g., by type of surgery or probiotic strain) could be beneficial.

Our subgroup analyses revealed critical insights into the optimal use of these regimens. The most important finding of our analysis is the statistically significant decrease in postoperative infections observed with multi-strain formulations containing both *Lactobacillus* and *Bifidobacterium* species (RR: 0.64, 95% CI: 0.49–0.83). This subgroup demonstrated near-zero heterogeneity, and meta-regression confirmed that strain type was a significant moderator of effect, explaining variabilities between studies. This suggests that the efficacy of probiotics in the perioperative setting is highly strain-specific, and multi-strain formulation may offer synergistic benefits in restoring gut barrier function and modulating immune responses compared to single-strain regimens (20, 21).

Furthermore, our analysis of clinical implications provides actionable data for surgical practice. The ARR for the multi-strain subgroup was 13.5% translating to an NNT of approximately 7. This means that treating 7–8 patients with this specific probiotic regimen would prevent one postoperative infection. Given the high morbidity and costs associated with postoperative infections, an NNT of 7 represents a highly favorable clinical and economic profile, especially considering the generally low cost and high safety profile of probiotics (22–24).

The analysis of regimen duration also provided interesting, albeit preliminary, insights. Shorter-duration regimens (≤7 days) showed a larger point estimate of benefit (RR: 0.48) and a more favorable NNT (∼5) compared to longer regimens (8–14 days). However, this finding was based on only two studies and requires further validation in larger trials. It is possible that a targeted, high-dose perioperative burst would be more effective than prolonged administration, but the optimal timing and duration remain to be definitively established.

The mechanism underlying the potential benefit is likely multifactorial, primarily related to the restoration of the gut-barrier function. Probiotics compete with pathogenic bacteria, produce antimicrobial substances, and modulate the host immune response by promoting anti-inflammatory pathways (25–28). This systemic effect is particularly important for decreasing remote infections, such as pneumonia, which were reported in some of the included studies. Its use can potentially decrease surgical stress response by increasing the production of short-chain fatty acids, which serve as energy sources for colonocytes and help maintain the tight junctions of the intestinal epithelium (29–32).

The significant heterogeneity found for the overall postoperative infective complications (*I*^2^ = 53%) highlights the need for careful interpretation and suggests that a single recommendation may not apply universally. The variability stems from several factors, including the type of surgery, the specific probiotic intervention and the control group used. While the colorectal surgery was the most represented in this meta-analysis, the inclusion of liver and pancreatic introduces clinical heterogeneity. Different surgical fields are associated with distinct levels of surgical stress and unique complication profiles. For instance, pancreatic surgery often involves a higher risk of infectious complications due to pancreatic leaks, which may not be as readily mitigated by probiotics as gut-derived infections (33). However, for colorectal surgery the gut microbials can be favorably altered by the use of probiotics. The included studies utilized a variety of probiotic formulations, ranging from single-strain (e.g., *Bifidobacterium bifidum*, *Saccharomyces boulardii*) to multi-strain combinations (Table 2). Our meta-analyses have suggested that multi-strain probiotics may offer superior efficacy compared to single strains, possibly due to synergistic effects (19, 34). The optimal dose and duration also remain undefined, with some studies employing only a short preoperative course while others extended administration into the postoperative period as well. Future research should prioritize head-to-head comparisons of different strains and dosing regimens. Two studies used “no intervention” as a control, while the rest used a placebo. The use of a true placebo is critical for maintaining blinding and minimizing performance bias. The inclusion of non-placebo-controlled studies, while necessary to maximize the data, may contributes to the observed heterogeneity.

The key strength of this meta-analysis is the rigorous adherence to the PRISMA guidelines and the use of the Cochrane RoB tool for quality assessment, which enhances the transparency and reliability of our findings. Furthermore, the inclusion of only RCTs provides the highest level of evidence. However, a primary limitation of this meta-analysis is the presence of an unclear risk of bias in several studies, particularly regarding allocation concealment and selective reporting. This is a common issue in surgical trials and underscores the need for improved reporting standards. The small number of included studies (*n* = 8) is another significant limitation, which restricts the statistical power of the analysis & precludes a formal assessment of publication bias. Also, the narrow crossing of the confidence interval for the overall POI [OR: 0.57 (95% CI: 0.32–1.01)] suggests that while the trend inclined towards probiotics, the finding is not statistically significant at the traditional *P* < 0.05 level, likely due to the limited sample size (*N* = 755). This necessitates caution and demands further large-scale, high-quality RCTs to confirm the significance of these findings (18, 35). Another potential limitation of our meta-analysis is that it may not have comprehensively captured studies using less common probiotic strains such as *Bacillus, Enterococcus, Streptococcus* or *Escherichia coli*. Future meta-analyses should explicitly include these strains in their search strategy to provide a more comprehensive assessment of the probiotic literature in surgical patients.

Despite these limitations, the robust signal observed in the multi-strain subgroup provides compelling evidence for the targeted use of specific probiotic formulations. Probiotics are generally safe and well-tolerated, making them a low-risk addition to Enhanced Recovery After Surgery (ERAS) protocols when the appropriate strains are selected (15, 36).

## 5 Conclusion

Our meta-analysis found a (narrowly missed) non-significant trend towards a reduction in postoperative infectious complications in patients undergoing major gastrointestinal surgery. However, the use of multi-strain *Lactobacillus* and *Bifidobacterium* formulations significantly decreases the risk of infectious complications following gastrointestinal surgery. With a highly favorable NNT of 7, these multi-strain regimens offer a clinically meaningful benefit. Further, large-scale, high-quality and methodologically robust RCTs are needed to definitively establish the efficacy of probiotics in this clinical setting and to determine the optimal probiotic strains, dosage and duration of treatment. The current evidence supports the targeted inclusion of multi-strain probiotics in ERAS protocols for patients undergoing major gastrointestinal surgery.

## Data Availability

The original contributions presented in the study are included in the article/Supplementary Material, further inquiries can be directed to the corresponding author/s.
